# The Identification of Novel Anti-Inflammatory Effects of Cannabigerol in the Kidney Tissue of Rats Subjected to a High-Fat High-Sucrose Diet

**DOI:** 10.3390/ijms26073114

**Published:** 2025-03-28

**Authors:** Anna Stepaniuk, Klaudia Sztolsztener, Karolina Konstantynowicz-Nowicka, Ewa Harasim-Symbor, Patrycja Bielawiec, Adrian Chabowski

**Affiliations:** Department of Physiology, Medical University of Bialystok, 15-089 Bialystok, Poland; klaudia.sztolsztener@umb.edu.pl (K.S.); karolina.konstantynowicz-nowicka@umb.edu.pl (K.K.-N.); ewa.harasim-symbor@umb.edu.pl (E.H.-S.); patrycja.bielawiec@umb.edu.pl (P.B.); adrian.chabowski@umb.edu.pl (A.C.)

**Keywords:** inflammation, diabetic kidney disease, cannabigerol, kidney, high-fat high-sucrose diet

## Abstract

The inflammatory state is a significant factor associated with diabetic kidney disease (DKD), making it one of the significant causes of chronic kidney disease. Despite the availability of data, there is a lack of targeted treatment strategies for diabetes-related kidney disorders. The aim of our study was to determine the impact of cannabigerol (CBG) on lipid precursors for inflammatory mediators during DKD development. A six-week experiment was conducted on male Wistar rats fed standard (Control) or high-fat high-sucrose (HFHS) diets. For the last 14 days of the experiment (5th and 6th weeks), half of the rats from the Control and HFHS groups intragastrically received CBG solution. Gas–liquid chromatography (GLC) was used to measure the activities of n-6 and n-3 polyunsaturated fatty acid (PUFA) metabolic pathways and the concentrations of arachidonic acid (AA), eicosapentaenoic acid (EPA), and docosahexaenoic acid (DHA) in selected lipid fractions. Immunoblotting was performed to assess the expression of proteins involved in the regulation of the inflammatory state. A multiplex immunoassay kit was used to determine kidney toxicity biomarker levels. Our results revealed that CBG administration to rats fed an HFHS diet decreased n-6 PUFA biosynthetic pathway activity in phospholipid (PL) and triacylglycerol (TAG) and increased n-3 PUFA biosynthetic pathway activity in TAG and free fatty acid (FFA). We also observed a reduction in the AA concentration in PL, FFA, and diacylglycerol (DAG). CBG supplementation reduced the level of kidney damage biomarkers, such as osteopontin (OPN). Our observations confirm that CBG has potential anti-inflammatory properties and may be successfully used for further research to seek targeted therapies of inflammatory disorders, including diabetic kidney disease progression.

## 1. Introduction

Recently, there has been a steady increase in the incidence of various metabolic abnormalities, which have been linked to increased mortality of the general population [1], including the components of central obesity, dyslipidemia, prediabetes, and diabetes conditions, which result from abnormal lipid and carbohydrate metabolisms [2]. Previous studies confirm that a sedentary lifestyle and a Western diet are major independent factors contributing to tissue dysfunction [3]. Moreover, Western type of diet has a strong connection with increased levels of protein carbonylation and lipid peroxidation products and an impairment in mitochondrial function, leading to enhanced reactive oxygen species (ROS) generation, thus related to oxidative stress occurrence [4]. The development of metabolic abnormalities is accompanied by the production of inflammatory molecules known as eicosanoids [5]. Eicosanoids are pro-inflammatory derivatives of 20-carbon polyunsaturated fatty acids (PUFAs) for which arachidonic acid (AA; 20:4 n-6) constitutes an early lipid precursor. Arachidonic acid is an omega-6 fatty acid (FA), which is released from membrane phospholipids (PLs) under the action of phospholipase A2 (PLA2) enzymes [6]. After its release from the membrane, it can be converted by three families of enzymes, including cyclooxygenase (COX), lipoxygenase (LOX), and cytochrome P450 (CYP450), to various metabolites, i.e., prostaglandin (PG) series 2 and leukotriene (LT) series 4 [7]. These mediators regulate the permeability of blood vessels, enabling the penetration of inflammatory cells, and they are also able to stimulate the activation of immune cells that contributes to an increase in the inflammatory response. In the pathogenesis of this inflammatory state, a key role is also played by nuclear factor κ B (NF-κB) expression, which regulates the increase in the generation and secretion of pro-inflammatory cytokines [8]. Thus, these molecules affect various cells and tissues, including the heart and kidneys, initiating a cascade of pro-inflammatory reactions that contributes to inflammatory processes, leading to structural and functional changes [9]. Current treatment options for kidney disease aimed at reducing inflammation include a range of medications that target both inflammation itself and its related complications [10]. Among these, the Renin–Angiotensin–Aldosterone System (RAAS) inhibitors, particularly Angiotensin Receptor Blockers (ARBs), have demonstrated anti-inflammatory effects in addition to their primary role in lowering blood pressure [11,12]. Similarly, Sodium-Glucose Cotransporter 2 (SGLT2) inhibitors are recognized for their protective effects on the kidneys and also show anti-inflammatory benefits, which can lead to better kidney health outcomes [11,13]. While some of these medications hold promise in lowering inflammatory markers, their overall effectiveness in enhancing clinical outcomes is still not well established. Although these treatments appear beneficial, existing evidence is limited when it comes to their ability to improve long-term kidney health.

Available data indicate that a prolonged supplementation of a diet rich in fats and glucose leads to an excessive accumulation of lipids in the kidney tissues, contributing to lipotoxicity. The enhanced availability of fatty acids and carbohydrates also increase the synthesis of pro-inflammatory mediators, initiating and accelerating the development of inflammation [14]. Moreover, an increased blood glucose concentration contributes to the glycation of the glomerular capillary basement membrane, and glomerular hyperfiltration results in the thickening of the basement membrane and increases its permeability to proteins, e.g., albumin [15]. Reduced renal clearance, occurring in chronic kidney disease (CKD), is responsible for higher levels of circulating cytokines, thus accelerating the development of inflammation and deteriorating renal functioning [16]. Recently, there has been considerable interest in compounds belonging to the phytocannabinoid family that constitute components of medical marijuana (Cannabis Sativa L.). One of the major phytocannabinoids, namely cannabigerol (CBG), is a safe and well-tolerated substance that does not produce intoxicating or adverse effects [17]. CBG has been shown to act as a moderate competitive antagonist of serotonin 1A receptor (5-HT1A) and binds weakly to cannabinoid type 1 and 2 receptors (CB_1_ and CB_2_). Available data confirm that a low dose of CBG exhibits antioxidant and anti-inflammatory properties, which results from the reduced activity of NF-κB and the phosphorylation of its inhibitor α (IκB- α), resulting in the decrease in cytokine generation and secretion, thereby decreasing the inflammatory state [18].

In the present study, we assess the impact of cannabigerol on the development of the low-grade inflammatory state in the kidney of rats fed a high-fat high-sucrose diet (HFHS) with particular emphasis on the activity of n-6 and n-3 PUFA biosynthetic pathways. We also determine the changes in the level of arachidonic acid, eicosapentaenoic acid (EPA; 20:5 n-3), and docosahexaenoic acid (DHA; 22:6 n-3) after CBG administration to rats subjected to an HFHS diet. Moreover, our study will allow the evaluation of the potential role of CBG in the therapy of the inflammatory state during kidney disorder with a focus on the expression of proteins regulating the synthesis of AA-derivatives and also the level of kidney toxic markers.

## 2. Results

### 2.1. The Influence of Cannabigerol on the Activity of the n-6 and n-3 Polyunsaturated Fatty Acid Metabolic Pathway and Ratio of n-6/n-3 Polyunsaturated Fatty Acid in the Kidney Tissue of Rats Subjected to a Standard Diet or High-Fat High-Sucrose Diet

In the kidney tissue, the n-6 polyunsaturated fatty acid (PUFA) metabolic pathway activity in phospholipid (PL) fraction was increased in rats treated with a high-fat high-sucrose diet alone and in the combination with cannabigerol (CBG) solution (HFHS: +16.3%, HFHS+CBG: +12.1%, *p* < 0.05, Table 1) in relation to the Control group. We also observed that the n-6 PUFA metabolic pathway activity in triacylglycerol (TAG) was increased in the HFHS group (HFHS: +27.3%, *p* < 0.05, vs. Control group, Table 1). In the same lipid pool, the n-6 PUFA metabolic pathway activity was decreased in the HFHS+CBG group (HFHS+CBG: −22.5% and −39.2%, *p* < 0.05, Table 1) than in the Control and HFHS groups, respectively. Additionally, we noticed that the n-6 PUFA metabolic activity of diacylglycerol (DAG) was decreased after the administration of CBG to rats fed an HFHS diet compared to the HFHS group (HFHS+CBG: −13.1%, *p* > 0.05, Table 1). We did not observe any notable alteration in the n-6 PUFA metabolic pathway activity in free fatty acid (FFA) pool (*p* > 0.05, Table 1). The activity of the n-3 PUFA metabolic pathway in PL was decreased in rats fed an HFHS diet (HFHS: −17.0%, *p* < 0.05, vs. Control group, Table 1). The n-3 PUFA of TAG was reduced in the HFHS+CBG group (HFHS+CBG: −27.7%, *p* < 0.05, vs. HFHS group, Table 1). We noticed no significant change in the n-3 PUFA metabolic pathway activity in DAG (*p* > 0.05, Table 1). Additionally, CBG administration to Control animals caused an enhancement in the n-3 PUFA in FFA pool (CBG: +22.8%, *p* < 0.05, Table 1).

In the kidney tissue, an increase in the ratio of n-6/n-3 PUFA was noticed in PL fraction under HFHS conditions (HFHS: +37.0%, *p* < 0.05, Table 1) compared to the Control group. In TAG, DAG, and FFA fractions, the ratio of n-6/n-3 PUFA did not significantly change (*p* > 0.05, Table 1).

### 2.2. The Influence of Cannabigerol on the Concentration of Arachidonic Acid in the Kidney Tissue of Rats Subjected to a Standard Diet or High-Fat High-Sucrose Diet

**Figure 1:**
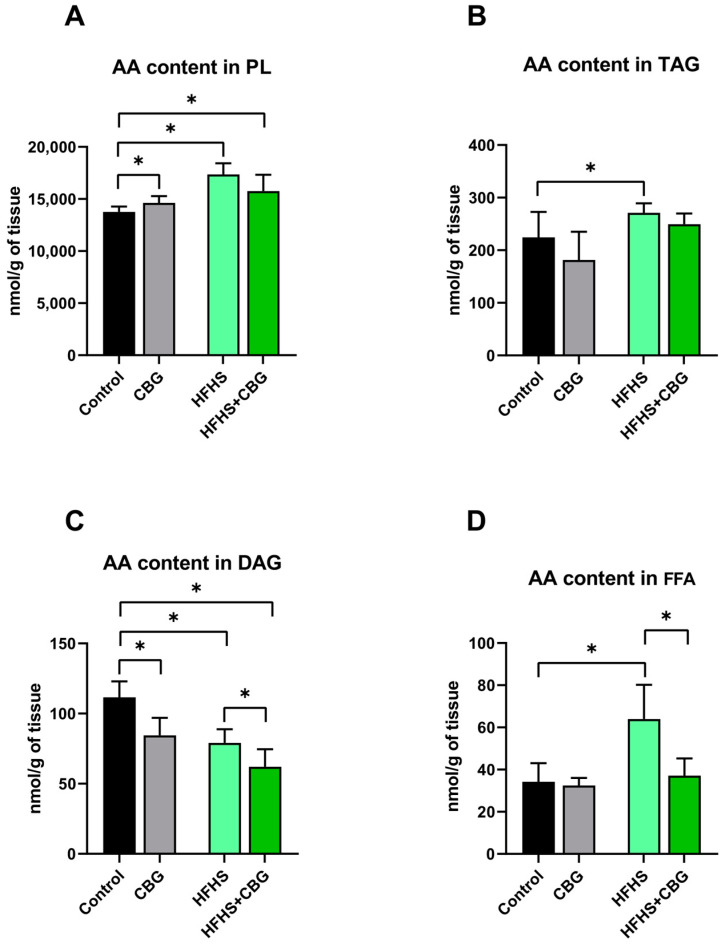
The arachidonic acid (AA) concentration in the examined lipid fractions, i.e., phospholipid (PL; (**A**)), triacylglycerol (TAG; (**B**)), diacylglycerol (DAG; (**C**)) and free fatty acid (FFA; (**D**)) in the kidney tissue of rats subjected to a standard diet (Control) or high-fat high-sucrose diet (HFHS) after two weeks of supplementation of cannabigerol (CBG) solution. The results are shown as the mean ± standard deviation (SD) and based on ten individual measurements in each experimental group. * *p* < 0.05—significant differences.

In PL fraction, the arachidonic acid (AA) content was higher in the rats from the CBG, HFHS, and HFHS+CBG groups (CBG: +6.4%, HFHS: +26.1%, HFHS+CBG: +14.5%, *p* < 0.05, Figure 1A) than in those from the Control group. In TAG, the content of AA was also increased in the HFHS group (HFHS: +18.2%, *p* < 0.05, vs. Control group, Figure 1B). The decrease in the AA level in DAG fraction was also noticed in all experimental groups (CBG: −24.3%, HFHS: −29.2%, HFHS+CBG: −44.4%, *p* < 0.05, vs. Control group, Figure 1C). In the same lipid fraction, the content of AA was decreased in the HFHS+CBG group (HFHS+CBG: −21.6%, *p* < 0.05, Figure 1C) in comparison with the HFHS group. Moreover, the AA concentration of FFA increased under HFHS conditions (HFHS: +42.2%, *p* < 0.05, vs. Control group, Figure 1D) and then reduced after CBG treatment (HFHS+CBG: −42.0%, *p* < 0.05, vs. HFHS group, Figure 1D).

### 2.3. The Influence of Cannabigerol on the Concentration of Eicosapentaenoic Acid in the Kidney Tissue of Rats Subjected to a Standard Diet or High-Fat High-Sucrose Diet

**Figure 2:**
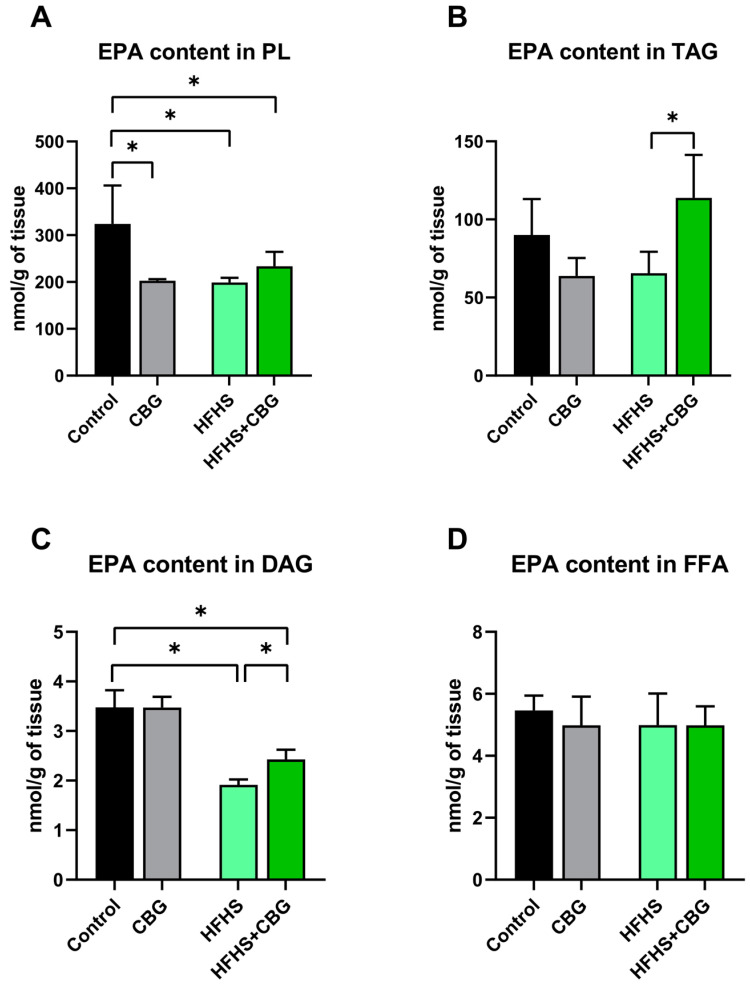
The eicosapentaenoic acid (EPA) concentration in the examined lipid fractions, i.e., phospholipid (PL; (**A**)), triacylglycerol (TAG; (**B**)), diacylglycerol (DAG; (**C**)) and free fatty acid (FFA; (**D**)) in the kidney tissue of rats subjected to a standard diet (Control) or high-fat high-sucrose diet (HFHS) after two weeks of supplementation of cannabigerol (CBG) solution. The results are shown as the mean ± standard deviation (SD) and based on ten individual measurements in each experimental group. * *p* < 0.05—significant differences.

In PL, the eicosapentaenoic acid (EPA) level was decreased in all examined groups (CBG: −37.4%, HFHS: −38.5%, HFHS+CBG: −28.0%, vs. Control group, *p* < 0.05, Figure 2A). In the HFHS+CBG group, the content of EPA in TAG pool was enhanced (HFHS+CBG: +44.0%, *p* < 0.05, Figure 2B) compared to the HFHS group. Additionally, we observed an increase in the EPA level of DAG in the HFHS+CBG group (HFHS+CBG: +45.7%, vs. HFHS group, *p* < 0.05 Figure 2C) and a decrease in the HFHS and HFHS+CBG groups (HFHS: −44.9%, HFHS+CBG: −30.2%, vs. Control group, *p* < 0.05 Figure 2C). Any significant change in the EPA content in FFA fraction was noticed (*p* > 0.05, Figure 2D).

### 2.4. The Influence of Cannabigerol on the Concentration of Docosahexaenoic Acid in the Kidney Tissue of Rats Subjected to a Standard Diet or High-Fat High Sucrose Diet

**Figure 3:**
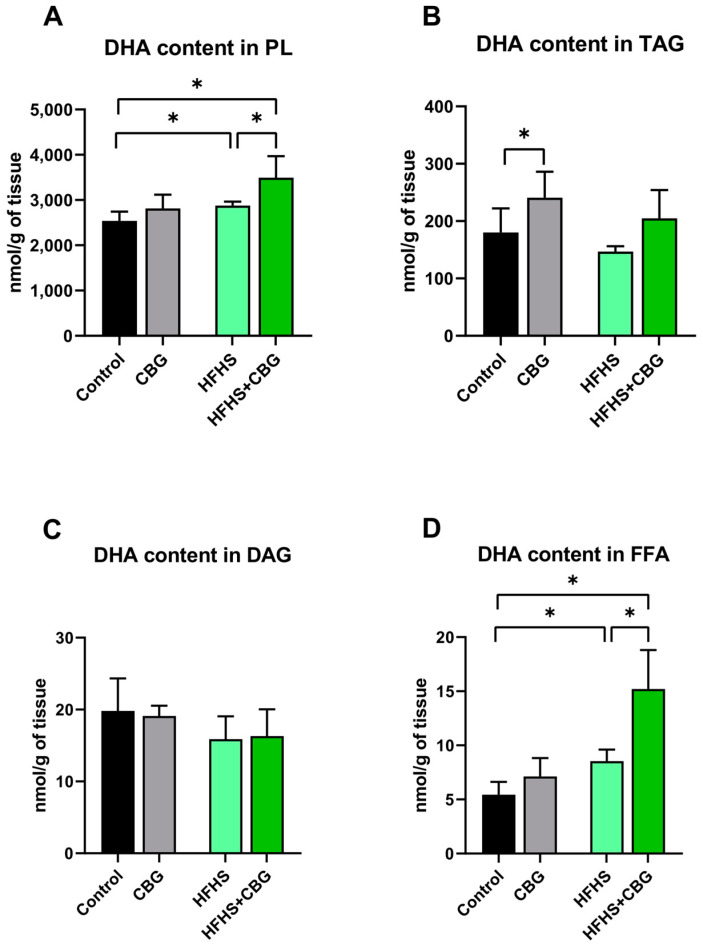
The docosahexaenoic acid (DHA) concentration in the examined lipid fractions, i.e., phospholipid (PL; (**A**)), triacylglycerol (TAG; (**B**)), diacylglycerol (DAG; (**C**)) and free fatty acid (FFA; (**D**)) in the kidney tissue of rats subjected to a standard diet (Control) or high-fat high-sucrose diet (HFHS) after two weeks of supplementation of cannabigerol (CBG) solution. The results are shown as the mean ± standard deviation (SD) and based on ten individual measurements in each experimental group. * *p* < 0.05—significant differences.

The content of docosahexaenoic acid (DHA) was increased in PL in rats fed an HFHS diet (HFHS: +13.3%, HFHS+CBG: +37.6% and +21.4%, *p* < 0.05, Figure 3A) in comparison with the Control and HFHS groups, respectively. CBG supplementation to the rats fed a standard diet resulted in an increment in the DHA content in TAG fraction (CBG: +33.7%, *p* < 0.05, vs. Control group, Figure 3B). In DAG fraction, the DHA level remained statistically unchanged (*p* > 0.05, Figure 3C). Additionally, we noticed an enhancement in the DHA content of FFA in rats fed an HFHS diet (HFHS: +55.9%, HFHS+CBG: +179.6% and +78.2%, *p* < 0.05, Figure 3D) compared to the Control and HFHS groups, respectively.

### 2.5. The Influence of Cannabigerol on the Expression of Proteins Regulating Arachidonic Acid Metabolism in the Kidney Tissue of Rats Subjected to a Standard Diet or High-Fat High-Sucrose Diet

**Figure 4:**
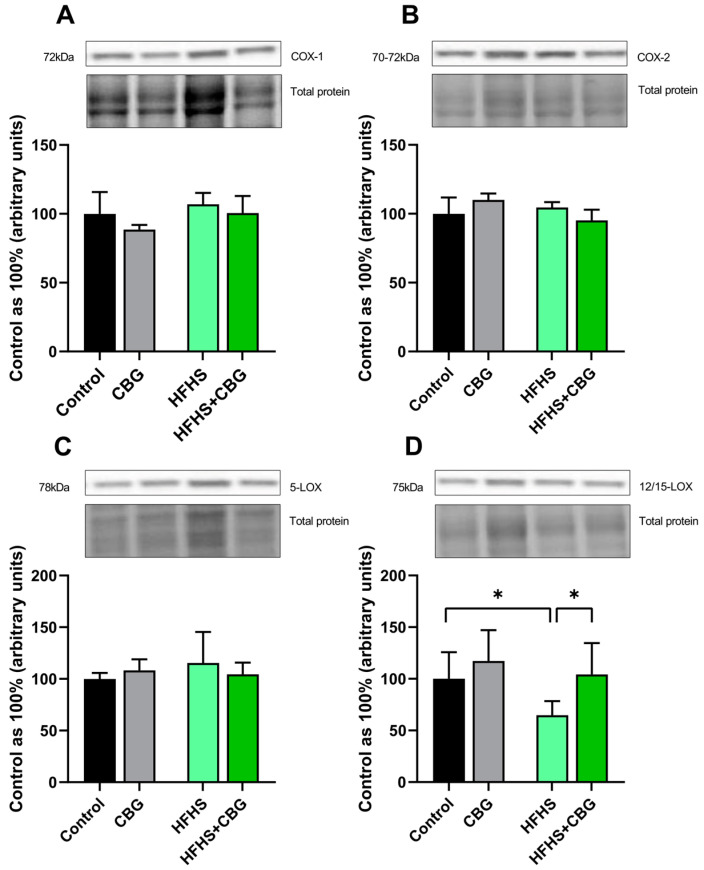
The expression of the proteins of cyclooxygenase 1 (COX−1; (**A**)), cyclooxygenase 2 (COX−2; (**B**)), 5-lipoxygenase (5−LOX; (**C**)), and 12/15-lipoxygenase (12/15−LOX; (**D**)) involved in arachidonic acid metabolism in the kidney tissue of rats subjected to a standard diet (Control) or high-fat high-sucrose diet (HFHS) after two weeks of supplementation of cannabigerol (CBG) solution. The results are shown as the mean ± standard deviation (SD) and based on six individual measurements in each experimental group. * *p* < 0.05—significant differences.

We observed no significant differences in the cyclooxygenase-1 (COX-1), cyclooxygenase-2 (COX-2), and 5-lipooxygenase (5-LOX) expressions (*p* > 0.05; Figure 4A, Figure 4B or Figure 4C, respectively) in all experimental groups. In the case of 12/15-lipooxygenase (12/15-LOX) expression, there was a reduction in the HFHS group (HFHS: −35.2%, *p* < 0.05, vs. Control group, Figure 4D), which was elevated after two weeks of CBG administration (HFHS+CBG: +61.0%, *p* < 0.05, vs. HFHS group, Figure 4D).

### 2.6. The Influence of Cannabigerol on the Expression of Other Proteins Involved in the Development of the Inflammatory State in the Kidney Tissue of Rats Subjected to a Standard Diet or High-Fat High Sucrose Diet

**Figure 5:**
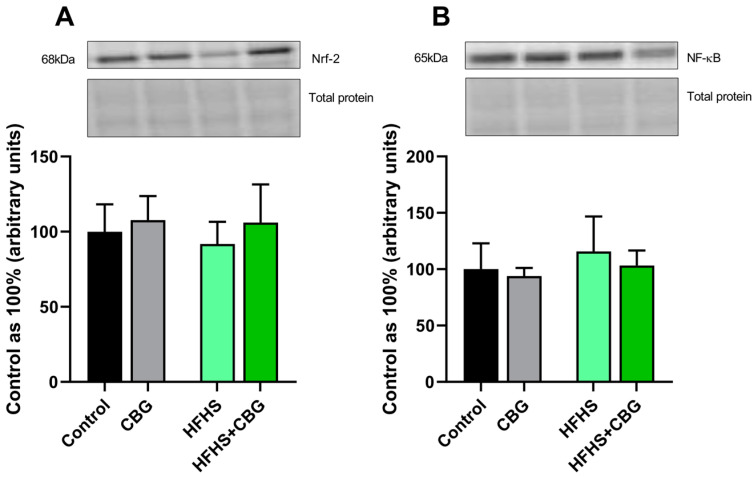
The expression of other proteins involved in the inflammatory response, i.e., nuclear factor erythroid 2-related factor 2 (Nrf−2; (**A**)) and nuclear factor κ B (NF−κB; (**B**)) in the kidney tissue of rats subjected to a standard diet (Control) or high-fat high-sucrose diet (HFHS) after two weeks of supplementation of cannabigerol (CBG) solution. The results are shown as the mean ± standard deviation (SD) and based on six individual measurements in each experimental group.

In all examined groups, the expression of nuclear factor erythroid 2-related factor 2 (Nrf-2) and nuclear factor κ B (NF-κB) was unchanged (*p* > 0.05; Figure 5A or Figure 5B, respectively).

### 2.7. The Influence of Cannabigerol on the Expression of Proteins Regulating the Fibrosis Process in the Kidney Tissue of Rats Subjected to a Standard Diet or High-Fat High-Sucrose Diet

**Figure 6:**
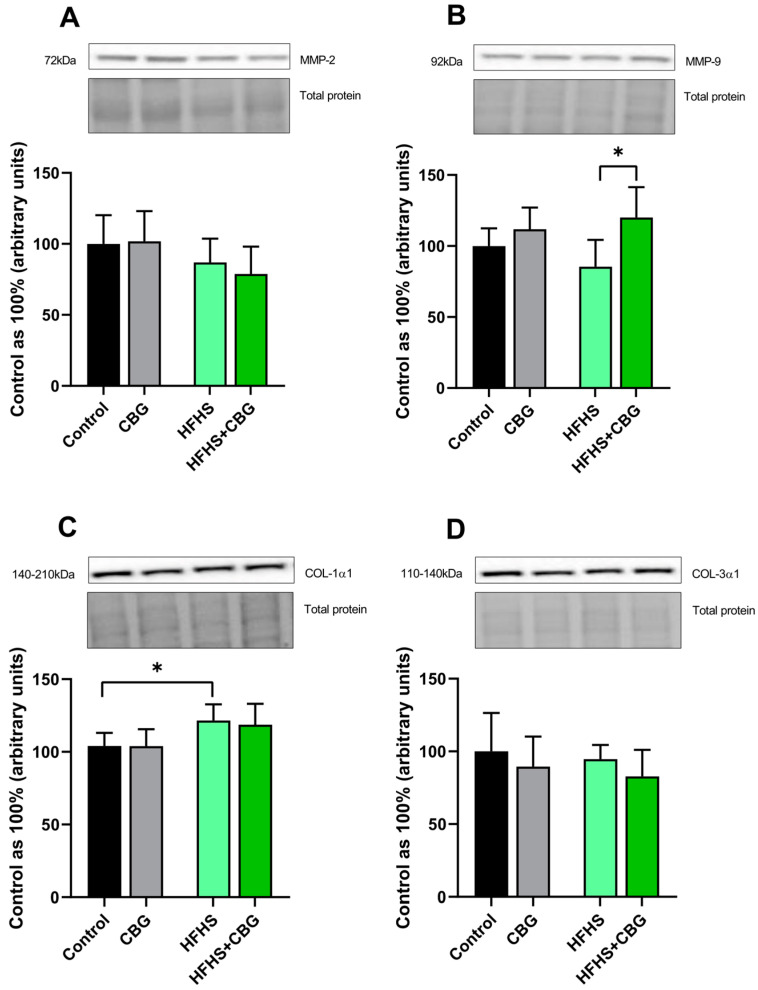
The expression of extracellular matrix proteins, i.e., matrix metalloproteinase 2 (MMP−2; (**A**)), matrix metalloproteinase 9 (MMP−9; (**B**)), collagen type 1 α 1 (COL−1α1; (**C**)), and collagen type 3 α 1 (COL−3α1; (**D**)) regulating fibrotic processes in the kidney tissue of rats subjected to a standard diet (Control) or high-fat high-sucrose diet (HFHS) after two weeks of supplementation of cannabigerol (CBG) solution. The results are shown as the mean ± standard deviation (SD) and based on six individual measurements in each experimental group. * *p* < 0.05—significant differences.

The expression of matrix metalloproteinase 2 (MMP-2) and collagen type 3 α 1 (COL-3α1) was significantly unchanged (*p* > 0.05; Figure 6A or Figure 6D, respectively) in all experimental groups. In the HFHS+CBG group, the expression of matrix metalloproteinase 9 (MMP-9) was increased (HFHS+CBG: +23.1%, *p* < 0.05, vs. HFHS group, Figure 6B). Moreover, an HFHS diet caused an increase in collagen type 1 α 1 (COL-1α1) expression (HFHS: +16.9%, *p* < 0.05, Figure 6C) compared to the rats from the Control group.

### 2.8. The Influence of Cannabigerol on the Concentration of Kidney Toxicity Markers in Urine Samples of Rats Subjected to a Standard Diet or High-Fat High-Sucrose Diet

**Figure 7:**
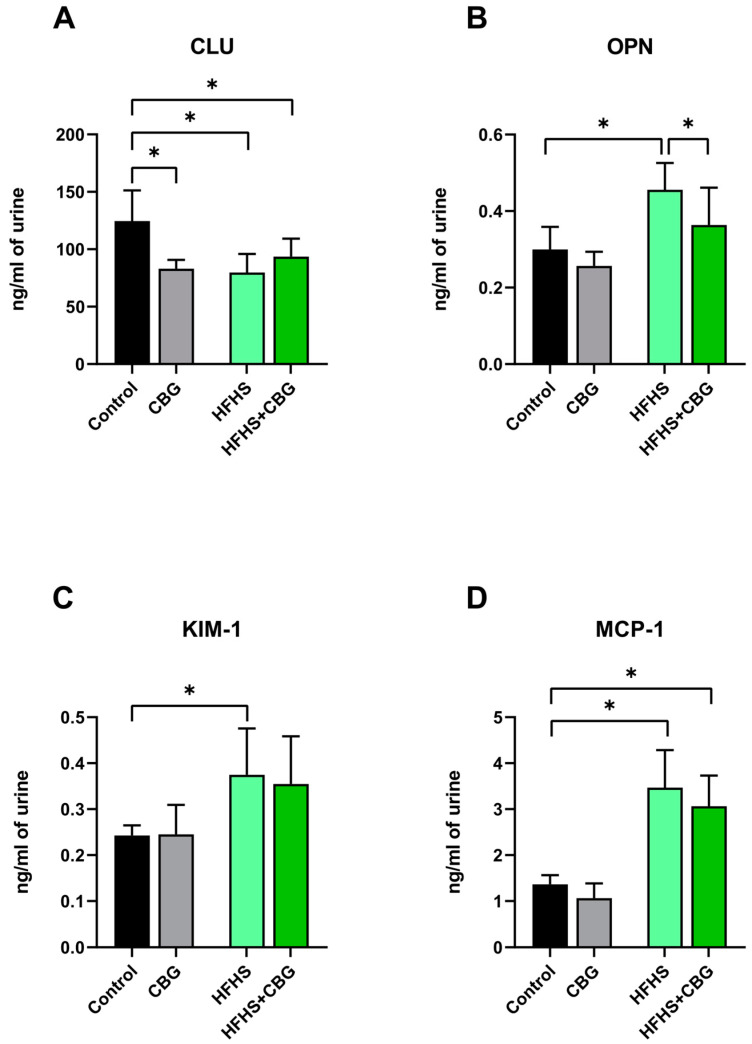
The concentration of kidney toxicity markers, i.e., clusterin (CLU; (**A**)), osteopontin (OPN; (**B**)), kidney injury molecule 1 (KIM−1; (**C**)), and monocyte chemoattractant protein 1 (MCP−1; (**D**)) in urine samples of rats subjected to a standard diet (Control) or high-fat high-sucrose diet (HFHS) after two weeks of supplementation of cannabigerol (CBG) solution. The results are shown as the mean ± standard deviation (SD) and based on ten individual measurements in each experimental group. * *p* < 0.05—significant differences.

The concentration of clusterin (CLU) was lower in urine samples in the rats from each experimental group (CBG: −33.4%, HFHS: −36.0%, HFHS+CBG: −24.9%, *p* < 0.05, vs. Control group, Figure 7A). We also noticed that a high-caloric diet caused an enhancement in the osteopontin (OPN) concentration (HFHS: +21.3% vs. Control group, *p* < 0.05, Figure 7B). Importantly, cannabigerol supplementation to rats fed an HFHS reduced the OPN level (HFHS+CBG: −20.2%, *p* < 0.05, Figure 7B) in relation to the HFHS group. In the HFHS group, the kidney injury molecule 1 (KIM-1) content was also increased (HFHS: +54.6%, *p* < 0.05, vs. Control group, Figure 7C). In comparison with the Control group, the concentration of monocyte chemoattractant protein 1 (MCP-1) was increased in both the HFHS and HFHS+CBG groups (HFHS: +154.4%, HFHS+CBG: +124.9%, *p* < 0.05, Figure 7D).

## 3. Discussion

A high-fat high-sucrose diet significantly impacts kidney homeostasis, leading to various pathological changes and functional impairments. Research shows that a diet rich in fat and sugar increases oxidative stress, inflammation, and structural damage in kidney tissues, ultimately contributing to CKD. Rodent studies have demonstrated that an HFHS diet can induce nephrotoxicity, marked by persistent damage to the kidney tissues and notable changes in histopathology [19]. The high intake of sucrose activates enzymes in the polyol pathway, resulting in a rise in free radical production, which adversely affects kidney function. Excessive carbohydrate consumption, especially from sucrose, contributes to insulin resistance and disturbances in renal function [20]. In rats fed an HFHS diet, elevated markers of oxidative stress and inflammation reveal a disruption in the balance between oxidants and antioxidants. Moreover, HFHS diet is linked to metabolic disorders like obesity and diabetes, which further compromise kidney function through mechanisms such as ectopic fat deposition and lipotoxicity [21]. It is important to search for a targeted therapeutic agent that significantly limits lipid deposition, with particular emphasis on the content of lipid precursors for inflammatory mediators in the course of diabetic kidney disease (DKD). Due to the potential anti-inflammatory effects of cannabigerol, the aim of our study was to assess the influence of this phytocannabinoid on the development of the inflammatory state in the kidney dysfunction induced by a high-fat high-sucrose diet.

The balance between n-6 and n-3 PUFA plays a significant role in maintaining the body’s homeostasis, and their impact on inflammation, which is closely related to the suppression of pro-inflammatory cytokine production for protecting against chronic diseases. The balance between n-6 and n-3 PUFA is crucial for regulating gene expression and the production of lipid mediators and cytokines, which can counteract the pro-inflammatory effects of AA-derived eicosanoids [22]. In our study, we assessed the effect of CBG and HFHS administration on the n-6/n-3 PUFA ratio in various lipid fractions, including phospholipid, triacylglycerol (TAG), diacylglycerol (DAG), and free fatty acid (FFA). We observed an increase in the n-6/n-3 PUFA ratio in PL fraction in rats from the HFHS group. As previously described by Voggel et al., in diabetic nephropathy, an increased ratio of n-3/n-6 PUFA reduces pro-inflammatory phospholipids and decreases the concentration of AA metabolites in the kidney tissue that mediate the occurrence of pro-inflammatory and prohypertensive effects [23]. CBG supplementation to rats subjected to an HFHS diet did not significantly affect the ratio of n-6/n-3 PUFA. Based on previous studies showing dose-dependent neuroprotective and anti-inflammatory properties of CBG in the rodent model, we suppose that the lack of significant changes in our results may be a consequence of the higher concentration of this agent used to conduct experiments [24,25]. We also observed that the administration of the HFHS diet contributed to an enhancement in the n-6 PUFA biosynthetic pathway activity in PL and TAG fractions. A higher level of n-6 PUFA is associated with an increased generation of pro-inflammatory mediators [26], such as interleukin 6 (IL-6), which may accelerate the deterioration of renal disease [27]. The aforementioned change is consistent with a study conducted by Bielawiec et al., in which a high-fat diet (HFD) enhanced the level of n-6 PUFA in lipid fractions such as FFA, PL, and TAG in skeletal muscle, increasing the pro-inflammatory response and oxidative stress [28]. Research indicates that a high level of n-6 PUFA is linked to a greater susceptibility to ischemic renal injury. It highlights that arachidonate metabolism, often associated with n-6 PUFA, contributes to renal inflammation through eicosanoids production, influencing leukocyte interactions and inflammatory responses [29]. n-6 PUFA may also influence renal inflammation by altering intercellular communication through connexins and pannexin, leading to kidney dysfunction [30]. In this study, CBG administration reduced the n-6 PUFA metabolic pathway activity in TAG and DAG fractions in rats with diabetic kidney disease induced by an HFHS diet. A decline in the n-6 PUFA biosynthetic pathway may modulate inflammatory gene expression with a progressive impairment in the generation of pro-inflammatory mediators, finally leading to the development of kidney inflammation following HFHS chow [31]. Among the family of PUFA, AA, EPA, and DHA play a significant role in the regulation of inflammatory processes [32]. In the present study, an HFHS diet reduced EPA content in PL and DAG and enhanced DHA content in PL and FFA. In the context of kidney disease, increasing the n-3 PUFA, EPA, and DHA levels or maintaining them at unchanged levels has renoprotective and anti-inflammatory properties [23]. It is noteworthy that CBG in the combination with HFHS diet resulted in an increase in the EPA concentration in TAG and DAG and an increase in the DHA content in PL and FFA. It is known that EPA and DHA can have a beneficial influence in inflammatory disease by reducing the production of prostaglandin E2 (PGE2), thromboxane A2 (TXA2), and leukotriene B4 (LTB4), which are responsible for inducing and worsening the inflammatory state [33]. Specifically, the supplementation of n-3 PUFA ameliorates proteinuria in diabetic nephropathy [34]. We hypothesize that CBG, by increasing the concentration of EPA and DHA as precursors for anti-inflammatory mediators, may lead to the limitation of the inflammatory response in the kidney of rats fed an HFHS chow. In our study, we also observed that the HFHS diet caused an increase in the AA concentration in PL, TAG, and FFA. Similar results regarding increase in the arachidonic acid concentration after HFD consumption were shown in Liu et al.’s study, in which the experimental diet promotes an increase in the systemic serum content of AA, especially in TAG fraction and n-6 PUFA in PL fraction [35]. The elevation in arachidonic acid level suggests the intensification of eicosanoids synthesis pathway that closely associates with the development of inflammatory processes in the kidney tissue [7,36]. Increased AA concentration across lipid fractions suggests that while AA levels raised in these three categories, there may be exceptions or variations in specific lipid subtypes or conditions. This indicates a significant but not universal increase across all lipid species within these fractions. Elevated AA levels can indicate altered energy storage and metabolism, potentially linked to increased cardiovascular risk due to the association of certain triglyceride profiles with metabolic disorders. This may also reflect enhanced lipolysis or mobilization of fatty acids, which can impact systemic inflammation and metabolic health. While increased AA can have detrimental effects, it also plays role in cellular signaling and may be necessary for certain physiological functions, suggesting a complex balance in dietary fat intake and health outcomes. CBG supplementation alleviated the increase in the AA concentration in DAG and FFA pools under lipid and sugar overload conditions, which was noticed in our study. The in vitro study shows that AA exposure is able to induce an upregulation of transforming growth factor β (TGF-β) and collagen type 4 α 1 (COL-4α1) expression, compounds associated with renal fibrosis [37]. Moreover, AA enhances angiotensin II (AngII)-induced gene expression, thereby activating mechanisms responsible for renal damage. We suppose that the reduction in AA concentration may be reflected in a potential protective effect of CBG by limiting renal fibrosis and damage [38]. We focused our scientific attention on the metabolism of arachidonic acid as a precursor for mediators with the strongest pro-inflammatory properties, which is the most important for the pathogenesis of arterial hypertension and chronic kidney diseases [23,39]. Arachidonic acid is metabolized in two main metabolic pathways, cyclooxygenases and lipoxygenases. In the context of the kidney, the COX isoform 1 (COX-1), as the constitutive form of this enzyme, plays an important role in both protection and potential damage to renal tissue. Prostaglandins produced by COX-1 contribute to maintaining renal blood flow by influencing the glomerular filtration rate (GFR) and increase vascular permeability, which promotes the delivery of nutrients and oxygen to the renal tissue. During chronic inflammation, increased COX-1 activity may lead to unfavorable changes in the kidney. Pro-inflammatory prostaglandins increase vascular permeability, leading to edema and the excessive infiltration of immune cells. This, in turn, may contribute to ongoing damage to renal tissue—fibrosis—directly negatively impacting renal function and contributing to CKD [40]. In addition, COX-2 is a constitutive isoform and its expression increased in response to inflammatory factors such as pro-inflammatory interleukins or mitogens and is the main enzyme associated with the inflammatory process due to its important role in the synthesis of mediators from the AA-derived prostanoid family [41,42,43]. On the other hand, in the LOX metabolic pathway, arachidonic acid is converted into leukotrienes and lipoxins (LX), which have pro-inflammatory and also anti-inflammatory effects, respectively. In a different manner, LT and LX affect the course and intensity of inflammation development and influence in the immune response [44]. In the present research, the expression of proteins regulating AA metabolism like cyclooxygenase 1 and 2 (COX-1 and COX-2), and 5- and 12/15-lipooxygenase (5-LOX and 12/15-LOX) were assessed. We noticed no significant changes in all the mentioned proteins under HFHS conditions. A recent analysis of kidney fragments stained with periodic acid–Schiff (PAS) indicated that a diet rich in fat induced an inflammatory state and exacerbated tubulointerstitial damage, glomerulosclerosis, and the number of local immunologic infiltrates. This alteration was observed only after 16 weeks of HFD feeding; thus, it may be a cause of the lack of notable changes in the COX and LOX expressions, indicating the initiation of kidney inflammation [43]. Nevertheless, in this study, an increase in the expression of 12/15-LOX was caused by the CBG administration to rats fed an HFHS. Increased 12/15-LOX expression, as a major anti-inflammatory isoform, enhanced lipoxin generation, which may suggest a rapid conversion of AA to lipoxin A4 (LXA4) or lipoxin B4 (LXB4). 12/15-LOX by LX can act on neutrophils and macrophages to exert an anti-inflammatory effect [45]. We suppose that, through the enhanced 12/15-LOX expression and possible LX synthesis, CBG inhibits pro-inflammatory leukotriene action by their synthesis and blocks receptors for them, as well as reducing leukocyte infiltration, finally leading to the reduction in the inflammatory process [7]. Previously, data indicated that lipoxins inhibit the growth in kidney/body mass ratio and also decrease glomerular dilation and matrix deposition in a diabetes mouse model [46,47]. The inflammatory state is also regulated by two major nucleus factors, NF-κB and nuclear factor erythroid 2-related factor 2 (Nrf-2). NF-κB is a transcription factor primarily involved in the regulation of gene expression associated with cytokines and chemokine synthesis and secretion. Both pathways, NF-κB and Nrf-2, can interact with forming a complex regulatory network that influences the inflammatory and oxidative responses. The increase in the NF-κB activity is associated with an intense inflammatory response and increased oxidative stress, whereas Nrf-2 contributes to a reduction in oxidative stress and an inhibition of NF-κB activity, thereby limiting the inflammatory response [48]. In our study, we did not observe any significant change in the expression of NF-κB and Nrf-2 in the both HFHS and HFHS+CBG groups. After being activated by stimuli such as oxidative stress and pro-inflammatory cytokines, NF-κB translocates to the cell nucleus, where it initiates the transcription of genes encoding inflammatory factors such as chemokines, e.g., monocyte chemoattractant protein 1 (MCP-1) and interleukins (TNF-α, IL-6, and IL-1β) [49]. This small discrepancy in our study may result from measurements conducted at different levels. Our data provided the protein level changes, and available research provided a change at the mRNA gene level, but the positive indicator confirming our hypothesis was the increase in the MCP-1 level in rats from the HFHS group. The pharmacological effects of CBG are primarily attributed to the activation of CB_1_ and CB_2_ receptors. Nonetheless, evidence indicates that the anti-inflammatory properties of these cannabinoids are mediated through the activation of peroxisome proliferator-activated receptor gamma (PPAR-γ), which have been shown to inhibit the synthesis of inflammatory cytokines, including TNF-α, IL-1β, and IL-6, in monocytes, while also suppressing macrophage activation in vitro [8]. Jeong et al. showed that CBG is able to inhibit JAK-STAT signaling that may play a role in alleviating inflammation and associated processes [50]. Further investigation is necessary to directly assess the influence of CBG on JAK-STAT signaling within this context, which is the major limitation of our study.

It has been reported that the occurrence of the infiltration of inflammatory cells into interstitial spaces is associated with renal fibrosis development due to the fact that cells, i.e., macrophages that secrete molecules with pro-inflammatory activity, as well as free radicals, lead to the production and deposition of collagen and other extracellular matrix (ECM) proteins, damaging epithelial and endothelial cells and promoting the fibrosis process [25]. Moreover, as previously published, the occurrence of dyslipidemia and renal inflammation accelerates fibrotic processes and could synergistically affect tubule function and increase renal damage [7]. Thus, we decided to examine the expression of the following selected parameters that regulate the deposition and turnover of ECM components: collagen type 1 α 1 (COL-1α1), collagen type 3 α 1 (COL-3α1), matrix metalloproteinase 2 (MMP-2), and matrix metalloproteinase 9 (MMP-9). In this experiment, an increase in COL-1α1 was caused by experimental feeding, suggesting the deposition of ECM components in the kidney tissue. Importantly, the administration of CBG to rats fed an HFHS diet resulted in an enhanced MMP-9 expression, which suggests that in the group of rats fed an HFHS diet, CBG increased the degradation of ECM proteins, thus pointing to its protective effect on the deposition of ECM components. Research conducted by Zhu et al. showed that an increased expression of MMP-9 resulted in an enhanced degradation of COL-4α1, thereby leading to cellular degradation of the basement membrane [51]. We suppose that CBG administration can inhibit the deposition of ECM proteins that limits the fibrotic processes and reveals the potential values of maintaining kidney function.

DAG is known to directly stimulate the activity of transient receptor potential channel C (TRPC), especially type 3, 6, and 7 cation channels, with a high permeability for Ca^2+^. The calcium-sensing receptor and its interactions with various cellular molecules are essential for regulating renal functions, including ion transport and podocyte development. The disruption of calcium signaling in podocytes can lead to impaired filtration barrier formation, highlighting its importance in kidney morphology and function and indicating a delicate balance that must be maintained for optimal renal health [52,53,54].

We also performed the measurement of kidney damage markers related to inflammation and fibrosis changes such as osteopontin (OPN), clusterin (CLU), kidney injury molecule 1 (KIM-1), and MCP-1 as well. In our study, there was a decrease in the CLU concentration and an increase in the OPN, KIM-1, and MCP-1 concentrations in the HFHS group. CLU protein functions have been well studied and indicate the ability of lipid transport and the clearance of misfolded proteins, and more importantly, the inhibition of the complement system and cell death [55]. Thus, its depletion can induce kidney functioning damage, including disturbances in glomerular filtration [56]. Next, data revealed that enhanced OPN content accelerates the deposition of extracellular matrix proteins, contributing to scar kidney tissue formation and finally leading to the loss function [57]. In patients with type 2 diabetes, elevated levels of OPN are linked to microvascular complications, including DKD. There is a positive correlation between OPN levels and the albumin–creatinine ratio (ACR), as well as with inflammatory markers such as C-reactive protein. Furthermore, high OPN levels have been associated with increased mortality rates among patients with chronic kidney disease, suggesting its potential as a prognostic indicator [58,59]. Although OPN shows promise as a biomarker for DKD, it is important to note that not all studies agree on its predictive value. Therefore, additional research is essential to fully establish its clinical utility. The increased concentration of other proteins, KIM-1 and MCP-1, responds to renal tubule damage and activates monocytes and macrophages to sites of inflammation, respectively [60,61]. Consequently, a diet enriched in fat and sugar may enhance the content of pro-damage renal factors, finally increasing inflammation, cell death, and interstitial fibrosis in diabetic kidney disease [23]. In our study, the supplementation of CBG to obese rats caused a decrease in the OPN concentration. Several studies described that the inhibition of OPN levels attenuates kidney injury and improves renal function [57,62]. The deletion of OPN reduced albuminuria and the expression of TGF-β and glomerular collagen IV in diabetic mice [62]. Thus, the decreased OPN content in the urine by CBG treatment may limit glomerular damage and protect against the development of inflammation in kidney damage and its progression to pro-fibrotic stages, suggesting that CBG might serve as a therapeutic agent [63].

In summary, diabetic kidney disease and complications related to the development of inflammation in the kidney require the search for a new class of safe and effective therapeutic agents. New treatment strategies based, for example, on cannabigerol administration may prove to be a better option due to the lack of unpleasant effects. In our study, we demonstrated that CBG supplementation to rats fed an HFHS diet resulted in a decrease in the n-6 PUFA biosynthetic pathway activity in TAG and DAG fractions and a decrease in the n-3 PUFA metabolic pathway activity in TAG fraction. Additionally, we observed a significant decrease in the AA concentration in DAG and FFA, an increase in the EPA concentration in TAG and DAG, and an increase in the DHA concentration in PL and FFA after CBG supplementation under HFHS feeding. CBG administration also led to an increase in the expression of 12/15-LOX and MMP-9, and a decrease in the OPN concentration. These results suggest that cannabigerol may have potential anti-inflammatory effects and could be used as a therapeutic agent to support the treatment of inflammatory-related diseases. However, further studies are necessary to confirm the efficacy and safety of using cannabigerol in clinical practice and to understand its precise mechanism of action in treating inflammatory diseases.

## 4. Materials and Methods

### 4.1. Experimental Procedures

The experiment was carried out on male Wistar rats, initially weighing 70–100 g. All animals were kept in plastic cages, two rats per cage, under standard holding conditions, i.e., a temperature of 22 ± 2 °C, a reverse light–dark cycle of 12/12 h, and access to standard rodent chow and water ad libitum. After an acclimatization period of 7 days, rats were randomly divided into four groups (*n* = 10): (1) Control group—animals fed with a standard diet containing energy values of 67% carbohydrate, 25% protein, and 8% fat (Labofeed B, Kcynia, Poland) with plain water; (2) CBG group—animals fed with a standard diet with plain water and cannabigerol (CBG; TargetMol, Wellesley Hills, MA, USA) supplementation; (3) HFHS group—animals fed with a high-fat diet (HFD) containing energy values of 60% fat, 20% carbohydrate, and 20% protein (Research Diet, New Brunswick, NJ, USA) with 20% (*w*/*v*) sucrose (Sigma Aldrich, Saint Louis, MO, USA) solution; and (4) HFHS+CBG group—animals fed with a high-fat diet with 20% (*w*/*v*) sucrose solution and cannabigerol supplementation. Cannabigerol solution was prepared in sesame oil (Oleofarm, Wroclaw, Poland) and then administered intragastrically to rats from the CBG and HFHS+CBG groups at a concentration of 30 mg/kg of body weight that was monitored weekly [64,65]. Additionally, rats from the Control and HFHS groups received only sesame oil by intragastric gavage. The administration of CBG solution and sesame oil was conducted once daily via gavage in the fifth and sixth weeks of experiment (for the last 14 days). At the end of the 6-week experiment, rats were anesthetized by intraperitoneal injection of phenobarbital (80 mg/kg of body weight). The kidney tissues were excised, cut in half, frozen at liquid nitrogen temperatures using a pre-cooled aluminum tong, and then stored at −80 °C for further measurements.

All procedures included in this experiment were performed in accordance with the regulations and guidelines of the Local Ethical Committee for Animal Experiments in Olsztyn (approval number: 19/2022).

### 4.2. Gas–Liquid Chromatography

Gas–liquid chromatography (GLC) was employed to determine the concentration of arachidonic acid (AA; 20:4 n-6), eicosapentaenoic acid (EPA; 20:5 n-3), and docosahexaenoic acid (DHA; 22:6 n-3) and to calculate the activity of n-6 and n-3 polyunsaturated fatty acid (PUFA) metabolic pathways and the n-6/n-3 PUFA ratio in selected lipid fractions, namely phospholipid (PL), triacylglycerol (TAG), diacylglycerol (DAG), and free fatty acid (FFA). Briefly, lipids were extracted from kidney tissues using a 2:1 (*v*/*v*) ratio of a chloroform and ethanol mixture as previously described by Folch et al. [66], with the addition of butylated hydroxytoluene and an internal standard like heptadecanoic acid. Following overnight incubation, water was added and then samples were centrifuged at 845× *g* for 10 min. The bottom layer of extract was collected and developed on silica plates intended for thin-layer chromatography (TLC) with the addition of resolving solution (heptane/isopropyl ether/acetic acid, 60:40:3, *v*/*v*/*v*). Separated PL, TAG, DAG, and FFA fractions were visualized under UV light and collected. Fractions were eluted using a mixture of diethyl ether and hexane (1:1, *v*/*v*), as well as a solution of chloroform, methanol, and water (5:5:1, *v*/*v*/*v*). The organic phase was then evaporated under a continuous stream of nitrogen. Following this, the transmethylation process was performed according to a method developed by Christie [67]. In brief, a diethyl ether solution combined with methyl acetate was added to the samples containing isolated TAG or PL and gently mixed. The lipids reacted with 1 M sodium methoxide in methanol at RT for 10 min. The reaction was stopped by introducing a saturated solution of oxalic acid in diethyl ether. After mixing samples, the solvent was evaporated again using a steady stream of nitrogen. For DAG, boron trifluoride in methanol solution was employed for methylation followed by incubation at 100 °C for 10 min. Pentane was then utilized to extract the fatty acid methyl esters (FAMEs), which were subsequently evaporated under nitrogen. All samples were dissolved in hexane. Analysis was conducted using a gas–liquid chromatograph (Hewlett-Packard 5890 Series II gas chromatograph; Agilent Technologies, Santa Clara, CA, USA) equipped with a flame ionization detector and a capillary column (HP-INNOWax GLC Column; Agilent Technologies, Santa Clara, CA, USA). The concentration of AA, EPA, and DHA in lipid pools was estimated and is expressed in nanomoles per gram of tissue. The activity of n-6 PUFA (20:4/18:2) and n-3 PUFA ((20:5 + 22:6)/18:3) metabolic pathways was calculated based on the fatty acid composition within specific lipid pools. Moreover, the n-6/n-3 PUFA ratio was calculated based on the activities of the n-6 and n-3 PUFA metabolic pathways in selected lipid fractions.

During gas–liquid chromatography, different control and standardization measurements were used. An internal standard (heptadecanoic acid) was added to quantitatively analyze the FA content. The retention times of FAME were used to report equivalent chain lengths of the saturated and unsaturated FA. The identification and further quantification of fatty acids were based on standard curves of each FA, which was prepared by dissolving each fatty acid to give a series of concentrations.

### 4.3. Immunoblotting

The Western blot technique was applied to assess the expression of proteins involved in the synthesis pathways of eicosanoid, specifically cyclooxygenase-1 (COX-1; Santa Cruz Biotechnology, Inc., Dallas, TX, USA), cyclooxygenase-2 (COX-2; Santa Cruz Biotechnology, Inc., Dallas, TX, USA), 5-lipoxygenase (5-LOX; Abcam, Cambridge, UK), and 12/15-lipoxygenase (12/15-LOX; Santa Cruz Biotechnology, Inc., Dallas, TX, USA); the expression of other proteins regulated inflammatory state development, specifically nuclear factor erythroid 2-related factor 2 (Nrf-2; Santa Cruz Biotechnology, Inc., Dallas, TX, USA) and nuclear factor κ B (NF-κB; Cell Signaling Technology, Inc., Danvers, MA, USA); and the expression of proteins regulated the extracellular matrix (ECM) composition, specifically matrix metalloproteinase 2 (MMP-2; Santa Cruz Biotechnology, Inc., Dallas, TX, USA), matrix metalloproteinase 9 (MMP-9; Santa Cruz Biotechnology, Inc., Dallas, TX, USA), collagen type 1 α 1 (COL-1α1; Santa Cruz Biotechnology, Inc., Dallas, TX, USA), and collagen type 3 α 1 (COL-3α1; Santa Cruz Biotechnology, Inc., Dallas, TX, USA). In brief, the kidney tissues were homogenized using an ice-cold radioimmunoprecipitation assay (RIPA) buffer with the addition of protease and phosphatase inhibitors (Roche Diagnostics, Mannheim, Germany). The homogenized tissue samples were kept under the incubation conditions at 4 °C for 45 min followed by centrifugation at 4 °C at 1000× *g* for 30 min. Then, supernatants were transferred to fresh tubes. In the supernatant fractions, the total protein concentration was assessed using a bicinchoninic acid (BCA) protein assay kit with the addition of bovine serum albumin (BSA) as a standard. After that, samples were diluted to an equivalent protein mass by the addition of Laemmli buffer (Bio-Rad, Hercules, CA, USA) and applied on Criterion TGX Stain-Free Precast Gels (Bio-Rad, Hercules, CA, USA). The proteins were separated during electrophoresis and subsequently transferred onto polyvinylidene fluoride (PVDF) or nitrocellulose membranes for semi-dry or wet transfer methods, respectively. Following the blocking step in Tris-buffered saline (TBS) with Tween detergent including 5% non-fat dry milk or 5% BSA, the membranes were immunoblotted overnight with a proper primary antibody. On the next day, the membranes were exposed to an appropriate secondary antibody conjugated with horseradish peroxidase (HRP). Subsequently, the protein bands were detected using a chemiluminescent Clarity Western ECL Substrate (Bio-Rad, Hercules, CA, USA). Densitometric measurements of obtained signals were conducted by a ChemiDoc visualization system (Image Laboratory Software; Bio-Rad, Hercules, CA, USA). The protein expression was standardized to the established total protein expression, which was set at 100% in the Control group.

### 4.4. Multiplex Immunoassay

The Bio-Plex Pro RBM Rat Kidney Toxicity (Panel 1) assay kit was used to measure the concentration of kidney damage markers in urine samples, i.e., clusterin (CLU), kidney injury molecule 1 (KIM-1), monocyte chemoattractant protein 1 (MCP-1), and osteopontin (OPN). At the beginning, all wells of the 96-plate were blocked by the addition of blocking buffer. After that, diluted urine samples (2×), standards, the control, and the blank were added to an appropriate well. Subsequently, beads were applied to each well of the assay plate, and then the plate was incubated for 1 h at RT. Following the series of washes, the addition of reconstituted detection antibodies, incubation (for 1 h at RT), and SA-PE dilution were carried out and the plate was then incubated (for 30 min at RT). Finally, the plate was washed three times and the beads were added. The 96-well assay plate was read on the Bio-Rad 200 System fitted with Bio-Plex Manager Software (Bio-Rad, Hercules, CA, USA).

### 4.5. Statistical Analysis

The experimental data are presented as the mean ± standard deviation (SD), which was based on ten independent measurements, except data from the immunoblotting technique (six independent measurements). Statistical analysis was performed using GraphPad Prism (software version: 8.2.1.; San Diego, CA, USA). The assumption of normality distribution and the homogeneity of variance were assessed using the Shapiro–Wilk test and Bartlett’s test, respectively. Statistical differences between experimental groups were examined using a two-way ANOVA (with Tukey’s test support) followed by an appropriate parametric *t*-test or non-parametric Mann–Whitney U test for variables with normal or non-normal distributions, respectively. For all data, a *p*-value < 0.05 was considered a statistically significant difference.

## Figures and Tables

**Table 1:** The n-6 and polyunsaturated fatty acids (PUFA) metabolic pathway activities and n-6/n-3 PUFA ration in the following lipid fractions: phospholipid (PL), triacylglycerol (TAG), diacylglycerol (DAG) and free fatty acid (FFA) in the kidney tissue of rats subjected to a standard diet (Control) or a high-fat high-sucrose diet (HFHS) after two weeks supplementation of cannabigerol (CBG) solution. The results are exhibited as mean ± standard deviation (SD) and based on ten individual measurements in each experimental group. * *p* < 0.05—significant differences compared to the Control group; # *p* < 0.05—significant differences compared to the HFHS group.

		Control	CBG	HFHS	HFHS+CBG
PL	n-6 PUFA	1.449 ± 0.076	1.556 ± 0.113	1.624 ± 0.061 *	1.684 ± 0.125 *
	n-3 PUFA	37.016 ± 5.335	40.438 ± 4.072	30.708 ± 2.953 *	33.529 ± 3.996
	n-6/n-3 PUFA	0.039 ± 0.004	0.040 ± 0.005	0.054 ± 0.006 *	0.048 ± 0.006
TAG	n-6 PUFA	0.054 ± 0.008	0.053 ± 0.006	0.069 ± 0.008 *	0.042 ± 0.005 * ^#^
	n-3 PUFA	0.798 ± 0.146	0.953 ± 0.155	0.819 ± 0.117	0.577 ± 0.096 ^#^
	n-6/n-3 PUFA	0.074 ± 0.007	0.062 ± 0.016	0.076 ± 0.003	0.075 ± 0.021
DAG	n-6 PUFA	0.574 ± 0.078	0.543 ± 0.009	0.655 ± 0.050	0.569 ± 0.044 ^#^
	n-3 PUFA	2.802 ± 0.245	2.642 ± 0.053	2.591 ± 0.589	2.450 ± 0.200
	n-6/n-3 PUFA	0.236 ± 0.013	0.233 ± 0.027	0.232 ± 0.026	0.196 ± 0.033
FFA	n-6 PUFA	0.419 ± 0.073	0.431 ± 0.040	0.491 ± 0.042	0.472 ± 0.078
	n-3 PUFA	1.322 ± 0.118	1.623 ± 0.098 *	1.547 ± 0.199	1.127 ± 0.252
	n-6/n-3 PUFA	0.326 ± 0.041	0.277 ± 0.029	0.296 ± 0.036	0.334 ± 0.078

## Data Availability

Data is contained within the article.
